# A case report of IgG4-related respiratory disease with pleural effusion and a literature review

**DOI:** 10.1097/MD.0000000000029338

**Published:** 2022-07-29

**Authors:** Qing Guo, Yue Ren, Quanyi Wang, Hongyun Pei, Shenghua Jiang

**Affiliations:** a College of Clinical Medicine, Jining Medical University, Jining, Shandong, China; b Pathology Department, Affiliated Hospital of Jining Medical University, Jining, Shandong, China; c Department of Respiratory Medicine, Affiliated Hospital of Jining Medical University, Jining, Shandong, China.

**Keywords:** diagnosis, immunoglobulin G4, pleural effusion, thoracoscope

## Abstract

**Rationale:**

IgG4-related respiratory disease (IgG4-RRD) is a chronic autoimmune disease that affects the respiratory system and organs outside the respiratory system. This study explored the diagnosis and treatment of a case of IgG4-RRD with unilateral pleural effusion diagnosed using medical thoracoscopy, and provides an associated literature review. This report summarizes the clinical characteristics of IgG4-RRD involving the pleura to improve the diagnosis of this disease.

**Patient concerns:**

A 39-year-old man presented with a 2-week history of cough and chest tightness. Both physical examination and imaging supported the presence of left pleural effusion.

**Diagnosis:**

Medical electronic thoracoscopy was performed to obtain a pleural biopsy, which showed lymphoplasmacytic infiltration, 40 IgG4+ plasma cells per High Power Field (HPF) on microscopy, IgG4/IgG ratio >50%, phlebitis obliterans, and storiform fibrosis. The final diagnosis was IgG4-RRD.

**Interventions and outcomes:**

The patient was treated with methylprednisolone, after which his symptoms improved, and he was discharged. Oral hormone therapy was continued outside the hospital. After 4 months, the patient returned to the hospital and his condition had improved significantly.

**Lessons:**

Pleural involvement in IgG4-RRD is rare, and its diagnosis depends on pleural biopsy. Thoracoscopy usually reveals pleural thickening, pleural nodules, and milky white plaques.

## 1. Introduction

IgG4-related disease (IgG4-RD) is a chronic systemic and progressive autoimmune disease involving multiple organs or tissues. It is characterized by an increase in serum IgG4 level, infiltration of tissue IgG4-positive plasma cells, and involvement of all tissues and organs in the whole body. IgG4-RD involving the respiratory organs and their accessory structures in the thoracic cavity is collectively referred to as IgG4-related respiratory disease (IgG4-RRD).^[1]^ IgG4-RRD involving the pleura is rare in clinical practice.^[2]^ Therefore, the present study analyzed the diagnosis and treatment process of a patient diagnosed with IgG4-RRD following medical thoracoscopy with unilateral pleural effusion as the major manifestation and associated with relevant literature review, so as to improve the understanding of IgG4-RRD and explore its diagnosis.

## 2. Case report

**Figure 1.**
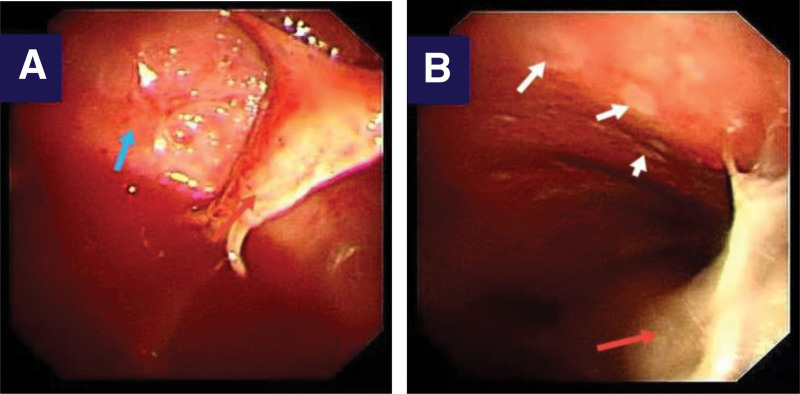
Under thoracoscopy, a large amount of light red effusion was observed in the thorax, with multiple matriform adhesions (red arrows in A and B), some of which were honeycomb, parietal and diaphragmatic pleura hyperemia and edema (blue arrows in A), milky white plaques (white arrows in B), thickened and uneven surface.

**Figure 2.**
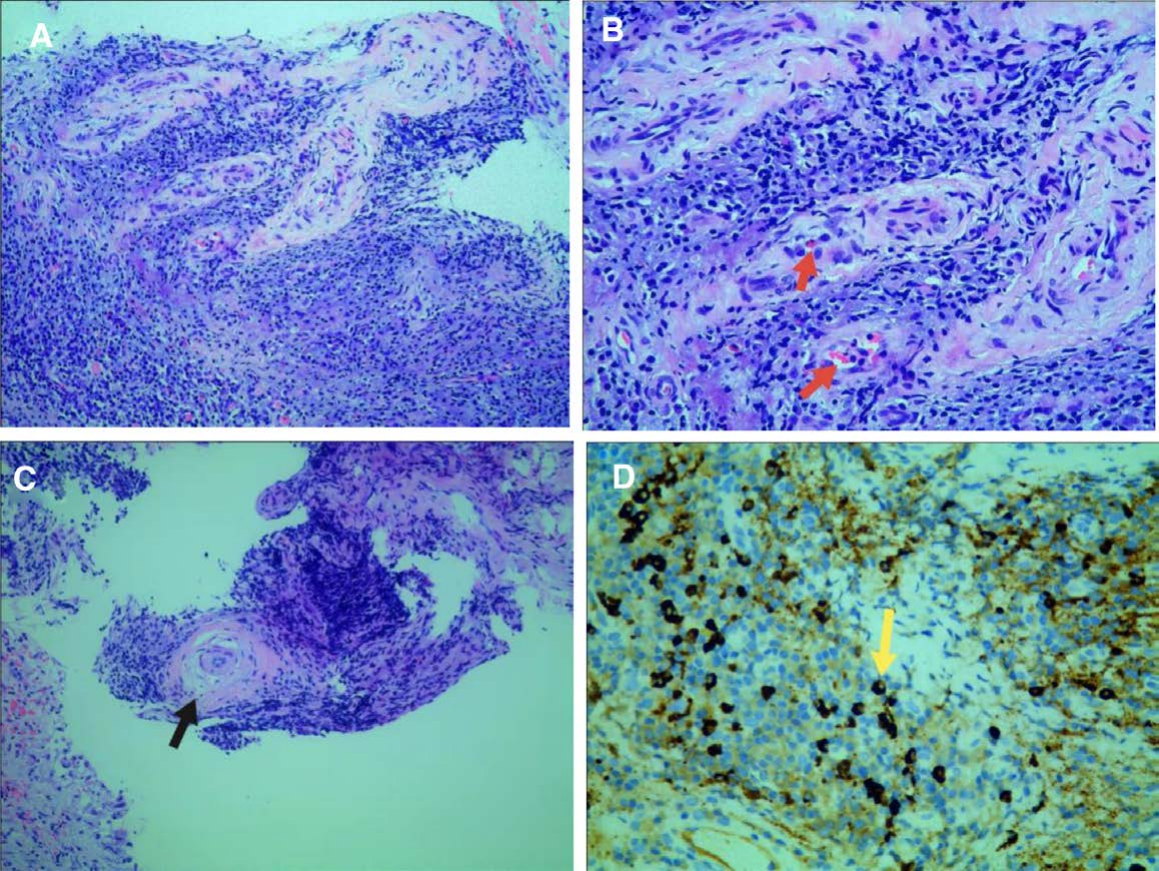
Pathology: Fibroconnective tissue with chronic inflammatory cell infiltration, lymphoid tissue hyperplasia and follicular formation, a large number of mature plasma cell infiltration with stromal fibroblast hyperplasia and hyaline degeneration can be seen in some areas (figure A HE, original magnification 100×). Phlebitis obliterans (red arrow in B HE, original magnification 200×); Matstriatal fibrosis (black arrow in C HE, original magnification 100×); Immunohistochemistry revealed 40 IgG4+ plasma cells (yellow arrow in D) per HPF, with an IgG4/IgG ratio greater than 50% (figure D Immunohistochemical staining, original magnification 400×).

**Figure 3.**
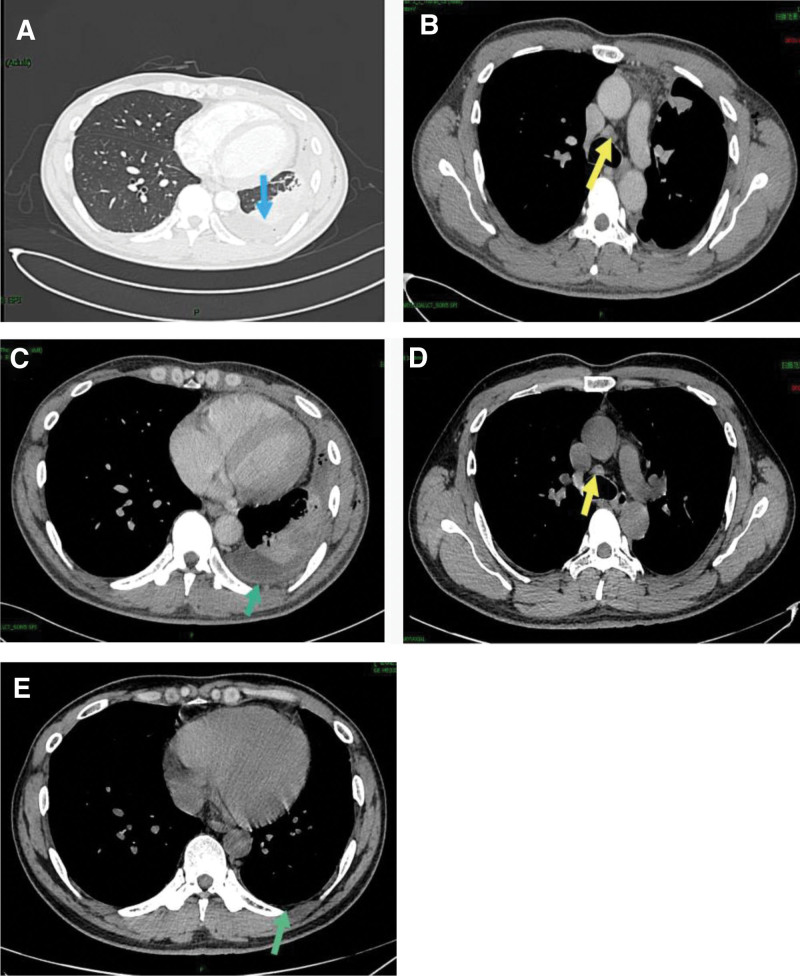
Enhanced chest and abdomen CT: left pleural effusion (blue arrow in A), enlarged lymph nodes in the mediastinum (yellow arrow in B), atelectasis; Pleural thickening at the left (green arrow in C); and changes in mediastinal lymph nodes and pleural at D and E after treatment.

A 39-year-old man, who had an otherwise healthy physical condition, was transferred to our hospital because of “cough and chest tightness for 2 weeks.” The patient was diagnosed with left pleural effusion at another hospital, with poor outcomes following conservative treatment. The patient underwent physical examination after admission, which found a body temperature of 37.6 °C, no enlargement of the superficial lymph nodes, low respiratory sounds on the left side, dullness on percussion, and no pleural friction sounds. The patient underwent further relevant investigations, which found a red blood cell count of 4.29 × 10^12^/L, white blood cell count of 8.49 × 10^12^/L, hemoglobin of 132 g/L, erythrocyte sedimentation rate of 92.00 mm/h, C-reactive protein of 97.00 mg/L, procalcitonin of 0.080 ng/mL, complement C3 of 2.09 g/L (normal range: 0.9–1.8 g/L), and complement C4 of 0.41 g/L (normal range: 0.9–1.8 g/L); antinuclear antibody quantitative determination, antinuclear antibody profile, anti-keratin antibody, and alpha fetoprotein results were all negative. *Mycobacterium tuberculosis*-specific T cells were not seen. The patient was provided underwent medical electronic thoracoscopy, which revealed a large number of light red effusions in the chest; multiple curtain-like adhesions with honeycomb-like appearance in some parts; congestion and edema of the parietal layer, diaphragm, and pleura; and uneven surface thickening. Multiple biopsies were taken from the parietal pleural lesions of the patient (Fig. 1A and B). Examination of the pleural effusion found pH 7.0, Rivalta test result of 2+, monocyte count of 0.95 × 10^6^/L, and specific gravity of 1.032. Biochemical analysis of the pleural effusion revealed a total protein of 53.1 g/L, lactate dehydrogenase of 371 U/L, glucose of 5.3 mmol/L, adenosine deaminase of 10.5 U/L. Investigation of tumor markers in the pleural effusion and ascites revealed a carcinoembryonic antigen of 4.26 ng/mL. In the left parietal pleural biopsy, pathological examination found fibrous connective tissue with chronic inflammatory cell infiltration, lymphoid tissue hyperplasia and follicular formation with the infiltration of a large number of mature plasma cells and interstitial fibroblast proliferation, hyaline degeneration, and storiform fibrosis in some parts, and phlebitis obliterans with the possibility of IgG4-related sclerosing disease in combination with immunohistochemical labeling results. The immunohistochemical results were: CD3 (+), CD20 (+), IgG (+), IgG4 (+), CK (−), κ (+), λ (+), CD21 (+), and Ki-67 (+, approximately 5%); and IgG4/IgG ratio >50% following pathological immunofluorescence investigation of the pleural biopsy sample, with 40 IgG4+ plasma cells per high power field (HPF) on microscopy (Fig. 2A–D). Serum IgG4 was determined to be 1.36 g/L quantitatively. Chest and abdomen enhanced computerized tomography (CT) revealed: 1. left pleural effusion, atelectasis, and left pleural thickening and 2. enlarged lymph nodes in the left supraclavicular area, mediastinum, left cardiophrenic angle, and hepatogastric space (Fig. 3A–C).

In our case, IgG4-RRD was diagnosed primarily based on the diagnostic criteria of respiratory involvement proposed in the seminar at the 54th Annual Meeting of the Japanese Respiratory Society (2014).^[1]^ The diagnostic criteria for IgG4-RRD include: (1) chest imaging changes including any of hilar/mediastinal lymphadenopathy, bronchial wall/bronchovascular bundle thickening, interlobular septal thickening, nodular shadow, infiltration shadow, pleural thickening, and/or pleural effusion; (2) increase in the concentration of serum IgG4 to >135 mg/dL;(3) tissues and organs in the thoracic cavity with >2 pathological features (a: n > 3; b: n = 2) of (i) a large number of lymphoplasmacytic infiltration around the bronchial vessels, interlobular septum, and/or pleura; (ii) IgG4+/IgG+ cell count >40% and/or >10 IgG4+ cells/HPF; (iii) phlebitis obliterans or arteritis obliterans; and (iv) storiform fibrosis or fibrosis consisting of spindle cells proliferating around infiltrating lymphocytes;(4) lesions in extrathoracic organs that meet the diagnostic criteria for IgG4-RD, such as sclerosing dacryocystitis/sialadenitis, autoimmune pancreatitis, IgG4-related sclerosing cholangitis, IgG4-related renal disease, and retroperitoneal fibrosis; and (5) hypocomplementaemia. A definite diagnosis was made if (1), (2), and (3)a or (1), (2), (3)b, and (4) were present; a definite diagnosis was made histologically if (1) and all 4 items of (3) were present. A highly possible diagnosis was considered if (1), (2), and (4) or (1), (2), (3)b, and (5) were present. Possible diagnosis was considered if (1), (2), and (3)b were present. CT examination of revealed pleural effusion and pleural thickening. Additionally, and increased concentration of serum IgG4 of 1.36 g/L; Pleural biopsy found: lymphoplasmacytic infiltration, 40 IgG4+ plasma cells per HPF on microscopy, an IgG4/IgG ratio >50%, phlebitis obliterans, and storiform fibrosis, which met the diagnostic criteria of (1), (2), and (3)a. The patient was confirmed to have IgG4-RRD after joint consultation and discussion between the Department of Respiratory Medicine, Department of Rheumatology and Immunology, and Department of Pathology. The patient was given methylprednisolone (40 mg, once a day) as treatment, and discharged from the hospital with improved symptoms. After discharge, the patient continued oral hormone treatment and maintained a stable condition during follow-up. After 4 months, the patient underwent hospital follow-up and chest computerized tomography (CT) examination; disappearance of the pleural effusion, significant reduction of the enlarged mediastinal lymph nodes (Fig. 3B vs D), and obvious improvement of the thickened pleura on the left side (Fig. 3C vs E) were seen.

## 3. Literature review

Ten patients with IgG4-RD involving the pleura^[3–12]^ were reported to have been diagnosed using medical thoracoscopy following literature review in China National Knowledge Infrastructure, the PubMed database, and Google Scholar. The patient we reported is the 11th case. The clinical data of these patients (Tables 1–3) are summarized below:

**Table 1:** Clinical characteristics of patients.

				Symptom						CRP (mg/L)		Author et al
Case	Age years/sex	Rapid or slow onset	Onset time	Dyspnea	Cough	Fever	Pain	Pleural effusion	Extrathoracic lesions		Serum IgG4 (g/L)	Year, refs
1	65/M	Unknown	Unknown	No	No	No	No	Right	PA, RP	10.8	2.99	Tamura K, 2020,^[3]^
2	43/F	Slow	3 months	Yes	No	No	No	Right	HP, HC	7.64	1.25	Tong X, 2017,^[4]^
3	46/M	Slow	1 year	Yes	No	No	No	Both	None	Unknown	1.42	Yasokawa N, 2020,^[5]^
4	78/M	Slow	2 years	No	No	No	No	Both	Leg edema, Bile ducts, CP	Unknown	7.60	Kondo T, 2016,^[6]^
5	78/M	Unknown	Unknown	Yes	Yes	No	No	Right	None	2.8	9.29	Saito Z, 2020,^[7]^
6	74/F	Slow	5 months	Yes	Yes	No	No	Both	Leg edema	Unknown	7.4	Ishida A, 2014,^[8]^
7	70/F	Unknown	Unknown	Yes	No	No	No	Left	Submandibular Sialadenitis		6.37	Shimada H, 2021,^[9]^
8	56/M	Rapid	15 days	Yes	No	No	Yes	Right	Pancreatitis	57.7	3.982	Damas F, 2019,^[10]^
9	65/M	SLOW	6 weeks	Yes	Yes	No	Yes	Right	None	10.7	2.53	Lococo F, 2019,^[11]^
10	63/M	Slow	10 weeks	Yes	Yes	No	No	Right	None	Unknown	2.84	Corcoran JP, 2015,^[12]^
11	39/M	Rapid	2 weeks	Yes	Yes	Yes	No	Left	None	97	1.36	

**Table 2:** Biochemical examination and pleural pathology of pleural effusion.

	Pleural biochemistry					Pathology histopathology				
Case	Exudate	Trabsudate	Main cell	ADA (U/L)	LDH (U/L)	Lymphop-lasmacytic infiltrate	Storiform fibrosis	Obliterative phlebitis	IgG4+ (HPF)	IgG4+/IgG+ (%)
1	Yes	No	Lymphocyte	46.6	112	Yes	No	No	10	22.4;50
2	Yes	No	Lymphocyte	Unknown	Unknown	Yes	Unknown	Unknown	80	>40%
3	Yes	No	Lymphocyte	36.4	85	Yes	Unknown	Unknown	22	42
4	Unknown					Yes	No	Unknown	100	70
5	Yes	No	Lymphocyte	71.9	Unknown	Yes	Unknown	Unknown	10	40
6	Yes	No	Monocyte	Normal	Unknown	Yes	No	No	91	91
7	Yes	No	Lymphocyte	Rise	Unknown	Yes	Unknown	Unknown	102	41.4
8	Yes	No	Lymphocyte	Unknown	489	Yes	Yes	Unknown	Unknown	>50%
9	Unknown					Yes	Unknown	Unknown	70	>30
10	Yes	No	Unknown	Unknown	Unknown	Yes	No	On	73	>40
11	Yes	No	Monocyte	10.5	371	Yes	Yes	Yes	40	>50

**Table 3:** Thoracoscopic characteristics and treatment prognosis.

	Lymph node				
	Hilar lung	Mediastinum	Thoracoscopic manifestations	Treatment and response	Refs
1	No	Yes	Mild pleural thickness with white and dense granular lesions, hypervascularization, and redness	PSL/effective	^[3]^
2	Unknown	Unknown	Multiple nodules, thickness and redness, a reduction in liver volume	Prednisone/effective	^[4]^
3	No	No	Blister-like nodules	PSL/effective	^[5]^
4	Unknown	Unknown	Milky white pleural plaques	PSL+pericardiectomy/effective	^[6]^
5	No	Yes	Thickness	PSL/effective	^[7]^
6	No	No	Thickness and redness	TD+ Pleurodesis+pre dnisone/effective	^[8]^
7	Unknown	Unknown	Normal	PSL/effective	^[9]^
8	No	No	A diffffuse inflflammation and fifibrin deposits	Methylprednisolone/effective	^[10]^
9	No	Yes	Thickness	Prednisone/effective	^[11]^
10	No	No	Mobile mass lesions, diffuse pleural thickening	PSL/effective	^[12]^
11	No	Yes	Adhesions, thickening, hyperemia and edema	Methylprednisolone/effective	

Among the 11 patients, the male to female ratio was 8:3, with an average age of 61.5 years old. There were two cases of acute onset (the onset time <2 weeks) and nine cases of chronic onset. Symptoms included dyspnea after exercise (9/11), cough (5/11), fever (1/11), chest pain (1/11), and abdominal pain (1/11). Signs included pleural effusion (11/11), palpable superficial lymph nodes (0/11) and hepatosplenomegaly (0/11). Blood tests revealed an increase in CRP levels (normal range: 0–10 mg/L) (5/7) and increased serum IgG4 levels (10/11). Imaging examination revealed pleural effusion(11/11), enlargement of the hilar or mediastinal lymph nodes (4/8), and involvement of additional extrapulmonary organs (6/11). Pleural effusion examination found that the effusion was exudate (11/11), and predominated by monocytes/lymphocytes (9/9), increased in adenylate deaminase (ADA) [normal range: 35–50 U/L, critical value of 45 U/L] (3/6), and increase in lactate dehydrogenase (critical value of 200 U/L) (2/4). Diseased pleura manifestations under medical thoracoscopy: pleural thickening (7/11), milky white plaque (4/11), nodular change (3/11), adhesions (4/11), vascular proliferation (4/11), vascular congestion and edema, and inflammatory changes (6/11). Treatment and prognosis included hormone therapy (11/11) and a length of 2 weeks to 2 years from onset to final diagnosis, with an average of approximately 6 months.

## 4. Discussion and Conclusions

IgG4-RRD is a systemic autoimmune disease closely related to IgG4. The disease mainly involves the respiratory organs in the chest and their accessory tissues, and can be accompanied by the involvement of different extrathoracic tissues and organs, showing diverse and non-specific symptoms. Thus far, there is a rare report of patients with IgG4-RRD involving the pleura who presented with pleural effusion. Here, we reported a case of IgG4-RRD, and reviewed the literature on clinical cases of IgG4-RD involving the pleura presenting with pleural effusion to improve the understanding of the diagnosis of IgG4-RRD.

Our study summarized the clinical features of 11 cases of IgG4-RD involving the pleura. This disease commonly has a chronic onset commonly and shows a high incidence in middle-ages and elderly men, with an average age at onset of 61.5 years. It is characterized by diverse clinical manifestations, including intrathoracic manifestations such as dyspnea and cough more frequently, as well as the manifestations of pleurisy such as low fever and pain. Additionally, it may also progress to other tissues and organs (e.g., pancreas, heart, bile duct) outside of the respiratory system. Patients with IgG4-RD may present with pleural effusion and pleural thickening through imaging, as well as another image feature of mediastinal lymph node enlargement. Furthermore, the biochemical examination of the pleural effusion fluid typically identifies it as exudation, mainly lymphocytic exudate, which can be accompanied by an increase in ADA and lactate dehydrogenase, but relatively low level of the tumor marker CEA, and normal or increased of serum CRP. The increased of IgG4 serum level is an important but nonspecific index for the diagnosis of IgG4-RRD, which can be found in other diseases, such as tumors and chronic infection.^[13]^ Moreover, there may be no abnormality in the serum IgG4 level in patients with IgG4-RRD.^[4]^ In thoracoscopy, patients may have diffuse or local pleural thickening, pleural nodules; milky white pleural plaques, pleural vascular hyperplasia and congestion, and even pleural adhesion. Clinically, in the cases of unexplained pleural effusion, electronic thoracoscopy can be regarded as an important examination. The possibility of IgG4-RRD should be suspected when patients are identified to have pleural thickening, nodular changes, milky white plaques, vascular hyperemia, hyperplasia and inflammatory changes on microscopy.

The diagnosis of IgG4-RRD depends primarily on the pathological results of the pleural biopsy. Massive lymphoplasmacytic infiltration. phlebitis obliterans or arteritis obliterans; and storiform fibrosis are the major pathological changes seen in IgG4-RRD. Phlebitis obliterans was only found in the present case among the 11 cases we summarized. According to the literature review, Shota Okamoto reported 17 cases of IgG4-related pleural diseases diagnosed using pleural histopathology.^[2]^ The corresponding results revealed that there was only one case of phlebitis obliterans; the other 10 cases showed fibrosis on pathological examination, with two cases of storiform fibrosis. Both phlebitis obliterans and storiform fibrosis are rare in patients with IgG4-RRD. Meanwhile, the principle of individualization should be followed in the medical treatment of IgG4-RRD. Glucocorticoid is the first-line drug for treating patients with IgG4-RRD. Immunosuppressive therapy can be considered as an auxiliary strategy when there is poor disease control.^[14]^ The prognosis is good in most patients with IgG4-RRD.^[15]^

Furthermore, there is a need to differentiate IgG4-RRD from the following diseases:

(1) Tuberculous pleurisy: Tuberculous pleurisy is the most common cause of pleural effusion in China. Common symptoms include chest pain, hot flashes, night sweats and other symptoms of tuberculosis poisoning. In addition, patients may be found to have local thickening of the pleural cavity, encapsulated pleural effusion, extensive pleural adhesion, and calcification on CT.^[16]^ Under medical thoracoscopy, there may be pleural necrosis, diffuse miliary tubercles, single or multiple pleural nodules, congestion, edema, thickening and adhesion.^[17]^ Significantly, patients with IgG4-RRD involving the pleura have different thoracoscopic manifestations, without the appearance of symptoms of tuberculosis poisoning.
(2) Metastatic tumor of pleura: The possibility of the metastatic malignant tumor should be considered when there is a bloody pleural effusion. Malignant tumors are more common in middle-ages and elderly people over 45 years old, with clinical manifestations of chest pain, dyspnea, and weight loss. CT findings generally include thickening of the parietal pleura (>1 cm), nodular and peripheral pleural thickening, thickening of the mediastinal pleura, and destruction of multiple ribs.^[18]^ In addition, there may be specific changes in the exfoliative cytology of pleural effusion and on pathological examination.
(3) Malignant pleural mesothelioma: Most patients with malignant pleural mesothelioma have a history of occupational exposure to asbestos, mostly unilateral pleural effusion,^[19]^ and clinical manifestations of progressive dyspnea, chest pain and cachexia. The imaging findings generally are annular pleural thickening and involvement of the mediastinal pleura and pleural nodules. The differential diagnosis can be made in combination with the detection of markers, such as the serum or pleural mesothelin,^[20]^ calretinin and WT-1.^[21]^ Pleural biopsy and pathological examination may provide the major defining characteristics for differentiation.
(4) Lymphoma: Painless lymphadenopathy is generally the first symptom for most patients with lymphoma. Some lymphomas, especially inert lymphomas, can be detected based on elevated serum IgG4 levels,^[22]^ which makes it difficult to be differentiated from IgG4-RD. Clinically, a lymph node or pleural biopsy can be performed to achieve a diagnosis according to the pathological results.

To summarize, IgG4-RRD involving the pleura is rare clinically. Affected patients mainly have clinical symptoms of cough and dyspnea, and may have pleural effusion, pleural thickening, and mediastinal lymph node enlargement on imaging. Furthermore, these patients may also be characterized by exudative pleural effusion, and normal or increased ADA levels. It is difficult to diagnose IgG4-RRD based on clinical manifestations and imaging features, and through pleural effusion examination. Its final diagnosis depends on pathological examination. Findings in our study support that the possibility of IgG4-RRD should be considered in cases of pleural thickening, pleural nodules, and milky white plaques on medical thoracoscopy.

## Author contributions

All authors read and approved the final manuscript.

Collect and analyze pathological examination:Quanyi Wang.

Collected and summarized the data: Yue Ren and Hongyun Pei.

Data curation: Hongyun Pei, Quanyi Wang, Yue Ren.

Funding acquisition: Shenghua Jiang.

Writing – original draft: Qing Guo.

Writing – review & editing: Qing Guo and Shenghua Jiang.

## References

[1] Matsui S Yamamoto H Minamoto S. Proposed diagnostic criteria for IgG4-related respiratory disease. Respir Investig 2016;54:130–2. 10.1016/j.resinv.2015.09.002 26879484

[2] Okamoto S Tsuboi H Sato R. IgG4-related pleural disease with aortitis and submandibular glands involvement successfully treated with corticosteroid: case-based review. Rheumatol Int 2020;40:1725–32. 32206878 10.1007/s00296-020-04555-y

[3] Tamura K Suzuki M Ishii S. IgG4-related disease with elevated adenosine deaminase in pleural effusion diagnosed clinically using thoracoscopy under local anesthesia and FDG-PET-CT. Respir Med Case Rep 2020;30:101066. 32373457 10.1016/j.rmcr.2020.101066 PMC7193316

[4] Tong X Bai M Wang W. IgG4-related disease involving polyserous effusions with elevated serum interleukin-6 levels: a case report and literature review. Immunol Res 2017;65:944–50. 28710703 10.1007/s12026-017-8934-y

[5] Yasokawa N Shirai R Tanaka H. Thoracoscopic findings in IgG4-related pleuritis. Intern Med 2020;59:257–60. 31554752 10.2169/internalmedicine.3031-19 PMC7008034

[6] Kondo T Uehara T. Immunoglobulin G4-related disease with fibroinflammatory lesions in the pleura, bile ducts and pericardium. CMAJ 2016;188:972. 27325135 10.1503/cmaj.160186 PMC5026516

[7] Saito Z Yoshida M Kojima A. Characteristics of pleural effusion in IgG4-related pleuritis. Respir Med Case Rep 2020;29:101019. 32071856 10.1016/j.rmcr.2020.101019 PMC7016278

[8] Ishida A Furuya N Nishisaka T. IgG4-related pleural disease presenting as a massive bilateral effusion. J Bronchology Interv Pulmonol 2014;21:237–41. 24992134 10.1097/LBR.0000000000000082

[9] Shimada H Kato Y Okuda M. Pleuritis associated with immunoglobulin G4-related disease under normal thoracoscopic findings: a case report. J Med Case Rep 2021;15:241. 33926544 10.1186/s13256-021-02718-4 PMC8086152

[10] Damas F Ghysen K Gester F. IgG4-related pleural disease in a patient with a history of unknown origin acute pancreatitis: a case report and review of the literature. Acta Clin Belg 2019;74:465–8. 30618348 10.1080/17843286.2018.1564173

[11] Lococo F Di Stefano T Rapicetta C. Thoracic hyper-IgG4-related disease mimicking malignant pleural mesothelioma. Lung 2019;197:387–90. 30941506 10.1007/s00408-019-00224-5

[12] Corcoran JP Culver EL Psallidas I. A 63-year-old man with a recurrent right-sided pleural effusion. Thorax 2015;70:504–7. 25572598 10.1136/thoraxjnl-2014-206423

[13] Matsui S. IgG4-related respiratory disease. Mod Rheumatol 2019;29:251–6. 30474465 10.1080/14397595.2018.1548089

[14] Zhang XQ Chen GP Wu SC. Solely lung-involved IgG4-related disease: a case report and review of the literature. Sarcoidosis Vasc Diffuse Lung Dis 2016;33:398–406. 28079853

[15] Kang J Park S Chae EJ. Long-term clinical course and outcomes of immunoglobulin G4-related lung disease. Respir Res 2020;21:273. 33076916 10.1186/s12931-020-01542-6 PMC7574178

[16] Zhou S Zhao J Song X. Imaging manifestations of B-mode ultrasound combined with CT in tuberculouspleuritis patients and the diagnostic value. Exp Ther Med 2018;16:2343–8. 30186477 10.3892/etm.2018.6471 PMC6122442

[17] Kong XL Zeng HH Chen Y. The visual diagnosis of tuberculous pleuritis under medical thoracoscopy: a retrospective series of 91 cases. Eur Rev Med Pharmacol Sci 2014;18:1487–95. 24899607

[18] Jiang WR Han ZP Tang X. Diffusion-weighted imaging diagnostic algorithm in patients with suspected pleural malignancy. Eur Radiol 2021;31:1–10. 34047851 10.1007/s00330-021-08013-6 PMC8589770

[19] Sinha S Swift AJ Kamil MA. The role of imaging in malignant pleural mesothelioma: an update after the 2018 BTS guidelines. Clin Radiol 2020;75:423–32. 32081346 10.1016/j.crad.2019.12.001

[20] Bibby AC Tsim S Kanellakis N. Malignant pleural mesothelioma: an update on investigation, diagnosis and treatment. Eur Respir Rev 2016;25:472–86. 27903668 10.1183/16000617.0063-2016 PMC9487555

[21] Brims F. Epidemiology and clinical aspects of malignant pleural mesothelioma. Cancers (Basel) 2021;13:4194. 34439349 10.3390/cancers13164194 PMC8391310

[22] Ishizuka K Shikino K Yokokawa D. Follicular lymphoma with hepatic accumulation on FDG-PET/CT masquerading IgG4-related disease. Radiol Case Rep 2021;16:2886–9. 34401019 10.1016/j.radcr.2021.07.008 PMC8350010

